# Hospital-Acquired Anemia in Patients Hospitalized in the Intensive Care Unit: A Retrospective Cohort Study

**DOI:** 10.3390/jcm11143939

**Published:** 2022-07-06

**Authors:** Piotr F. Czempik, Dawid Wilczek, Jan Herzyk, Łukasz J. Krzych

**Affiliations:** 1 Department of Anaesthesiology and Intensive Care, Faculty of Medical Sciences in Katowice, Medical University of Silesia, 40-752 Katowice, Poland; kait@uck.katowice.pl; 2 Students’ Scientific Society, Department of Anaesthesiology and Intensive Care, Faculty of Medical Sciences in Katowice, Medical University of Silesia, 40-752 Katowice, Poland; s76509@365.sum.edu.pl (D.W.); s76420@365.sum.edu.pl (J.H.)

**Keywords:** anemia, blood loss, coagulation, hemoglobin, intensive care unit, transfusion

## Abstract

Hospital-acquired anemia (HAA) is prevalent in patients hospitalized in the intensive care unit (ICU). Iatrogenic blood loss (IBL) may aggravate existing anemia or lead to a need for red blood cell (RBC) transfusion. The aim of our study was to analyze hemoglobin (Hb) concentration changes in up to 14 days, as well as all potential sources of IBL, in consecutive patients admitted to the intensive care unit (ICU) in the years 2020–2021. Patients admitted due to bleeding were excluded. Anemia on admission was present in 218 (58.8%) patients—47 (48.9%) surgical and 171 (62.2%) non-surgical (*p* = 0.02). Gradual decrease in Hb was seen in all ICU patients. Eighty-one (21.8%) patients required RBC transfusion. The first unit of RBC was transfused on day 7 (IQR 2–13) and the second on day 11 (IQR 4–15) of ICU hospitalization. The median admission Hb in patients who required RBC transfusion was 10.2 (IQR 8.5–11.8) and, in those who did not require transfusion, it was 12.0 (IQR 10.2–13.6) g/dL (*p* < 0.01). Anemia on admission was associated with a need for RBC transfusion (*p* < 0.01). Average decrease in Hb during the first week of ICU hospitalization in patients with and without anemia on admission was 1.2 (IQR 0.2–2.3) and 2.8 (IQR 1.1–3.8) g/dL (*p* < 0.01), respectively. Percentage of patients who bled at the insertion site of invasive devices was as follows: percutaneous tracheostomy—46.7%, therapeutic plasma exchange (TPE) catheter—23.8%, dialysis catheter—13.3%, gastrostomy—9.5%, central venous catheter—7.8%. Moreover, circuit clotting occurred in 17.7 and 9.5% of patients undergoing dialysis and TPE, respectively. Median blood loss for repeated laboratory testing in our study population was 13.7 (IQR 9.9–19.3) mL per patient daily. Anemia is highly prevalent among medical and surgical patients on admission to ICU and is associated with RBC transfusion. Patients who required RBC transfusion had significantly lower daily Hb concentrations. Severity of disease did not seem to have impact on Hb concentration. IBL associated with invasive devices and extracorporeal therapies is frequent in ICU patients and may lead to a gradual decrease in Hb concentration. Further studies are required to analyze causes of HAA in the ICU.

## 1. Introduction

Anemia is prevalent in patients hospitalized in the intensive care unit (ICU) and may concern up to 66% of patients at admission [1] and almost all patients within the next 72 h [2]. In the prospective cohort study by Thomas et al., the incidence of anemia in the ICU was as high as 98% [3]. Anemia that develops as a consequence of hospitalization has been named hospital-acquired anemia (HAA). It has been reported that, generally, 75% of non-anemic patients develop HAA. Hospital-acquired anemia leads to the prolongation of hospital stay and increased mortality [4].

As virtually all patients being admitted to the ICU are anemic or at risk of anemia, the presence of anemia should always be sought for at admission to the ICU and throughout ICU hospitalization. One of the important causes of ICU HAA is iatrogenic blood loss (IBL) for laboratory testing [5,6]. Another cause could be episodes of bleeding in patients during hospitalization in the ICU. Insertion sites of vascular catheters and endotracheal tubes have been reported to be the most common sites of bleeding [7]. Clotting of the extracorporeal circuit of continuous renal replacement therapy (CRRT) apparatus could also lead to significant blood loss [8]. The accurate assessment of the causes of IBL in ICU patients is important for planning preventive measures in this vulnerable patient population.

The aim of our study was to analyze Hb concentration changes as well as the potential causes of IBL in patients hospitalized in the ICU. 

## 2. Materials and Methods

We performed a comprehensive retrospective analysis of all patients admitted to the ICU from January 2020 to December 2021 (*n* = 494). We excluded patients admitted due to major bleeding [9,10] and hemorrhagic shock (*n* = 114) and patients in whom Hb was not determined on admission to ICU (*n* = 9). We analyzed Hb concentration changes up to 14 days of ICU hospitalization. We divided patients into groups according to the presence of anemia on admission, as well as red blood cell (RBC) transfusion.

### 2.1. Setting

Our ICU is a 10-bed mixed surgical-medical unit located in a tertiary care teaching hospital. For continuous renal replacement therapy (CRRT), we solely used regional citrate anticoagulation (RCA), whereas, for the therapeutic plasma exchange (TPE) it was solely unfractionated heparin (UFH). We used standard therapeutic dosing of UFH and did not monitor the anticoagulant effect during the procedure. 

### 2.2. Patients Data

Demographic, clinical, and laboratory data were retrieved from electronic health records (AMMS, Asseco Medical Solutions, Rzeszow, Poland). We used the most recent definitions for sepsis and septic shock [11]. We defined acute kidney injury (AKI) according to Kidney Disease Improving Global Outcomes (KDIGO) initiative [12]. Acute liver injury (ALI) was defined as serum total bilirubin above local laboratory reference range (i.e., 1.2 mg/dL). We assessed patients’ severity of disease according to 3 established classification systems: Simplified Acute Physiology Score II (SAPS II), Acute Physiology and Chronic Health Evaluation (APACHE II), and Sepsis-related Organ Failure Assessment (SOFA). Severity of disease was assessed on admission to the ICU. In order to maximize accuracy of results, we used Hb concentrations derived from complete blood count (CBC) tests, which is considered the gold standard. We never used Hb results obtained from blood gas analysis or non-invasive methods. Anemia was defined as a Hb concentration below the World Health Organization reference range, i.e., 13.0 g/dL for men and 12.0 g/dL for women. We retrieved data on RBC transfusions. We retrieved patient data that could have impact on iatrogenic blood loss in our patients, including ICU length of stay (ICU-LOS) and the presence of organ failures that could potentially cause/aggravate blood loss (acute liver injury, acute kidney injury).

### 2.3. IBL Data

We retrieved data regarding: CRRT (duration, episodes of extracorporeal circuit clotting), dialysis catheters (number inserted in the ICU, episodes of bleeding at the insertion site), dialysis catheters inserted for TPE (number inserted in the ICU, episodes of bleeding at the insertion site), central venous catheters (CVC) (number inserted in the ICU, episodes of bleeding at the insertion site), percutaneous tracheostomies (Griggs method) (number inserted in the ICU, episodes of bleeding at the insertion site), data on percutaneous endoscopic gastrostomies (PEG) (number inserted in the ICU, episodes of bleeding at the insertion site), episodes of gastrointestinal bleeding (bloody or coffee-ground vomiting; melena; bleeding confirmed endoscopically). An episode of extracorporeal circuit clotting in our setting corresponded to an IBL of approximately 200 mL (circuit priming volume). In patients in whom both TPE and CRRT was performed, the episodes of dialysis catheter insertion and bleeding at the insertion site were accounted for in each group. We retrieved information on the number of test tubes collected for common laboratory analysis: arterial blood gas (volume 1.0 mL), biochemistry (volume 2.5 mL), complete blood count (volume 2.0 mL), blood culture (volume 10 mL), coagulation (volume 2.7 mL). Blood loss of approximately 100 mL corresponds to a decrease in Hb concentration by 0.2 g/dL, whereas transfusion of 1 unit of whole blood (app. 500 mL, Hct 0.36–0.44) usually increases Hb concentration by 1 g/dL.

### 2.4. Statistical Analysis

For the perform statistical analysis, we used MedCalc v.18 statistical software (MedCalc Software, Ostend, Belgium). The continuous variables were presented as a median and interquartile range (IQR). Mann–Whitney test and Student’s t-test were used to verify the intergroup differences for continuous variables. The categorical data were expressed as numbers and percentages. Statistical significance was established by the chi-squared or the Fischer exact tests. Associations between variables were tested using Spearman rank correlation analysis. We adopted statistical significance as level *p* < 0.05.

### 2.5. Ethics

Ethical review and approval were waived for this study due to retrospective observational manner of the study (PCN/0022/KB/258/19).

## 3. Results

We analyzed 371 patients. The characteristics of the study population are presented in Table 1. 

There was high prevalence of sepsis/septic shock and AKI in the study population. The study population had a high severity of disease according to SAPS II, APACHE II and, SOFA, with predicted mortality of 32.6%, 12%, and ≤33%, respectively. The selected laboratory parameters on admission to ICU in the study population are presented in Table 2.

Median Hb concentration for the whole group was 11.6 (IQR 9.8–13.3) g/dL. Majority of patients were anemic on admission to ICU (*n* = 218, 58.8%). There were 96 (25.9%) surgical (postoperative) and 275 (74.1%) non-surgical patients. In the first group, 47 (48.9%) were anemic, whereas, in the latter, it was 171 (62.2%) (*p* = 0.02). Anemic compared to non-anemic patients had lower RBC indices with the exception of RDW and RDW-SD (higher values) and MCV (no difference) (Table 2). As far as iron status markers are concerned, there were no differences between patients with and without anemia on admission to ICU with the exception of transferrin, which was lower in patients with anemia (*p* < 0.01). 

During ICU hospitalization, 81 (21.8%) patients received at least 1 unit of RBC. The total amount of RBC transfused was 253, and the median number of RBC per patient was 2 (IQR 1–4). The first unit of RBC was transfused on day 7 (IQR 2–13) and the second on day 11 (IQR 4–15) of ICU hospitalization. The number of patients who required RBC transfusion among patients with and without anemia on admission was 64 (29.3%) and 17 (11.1%), respectively. (*p* < 0.001).

**Figure 1:**
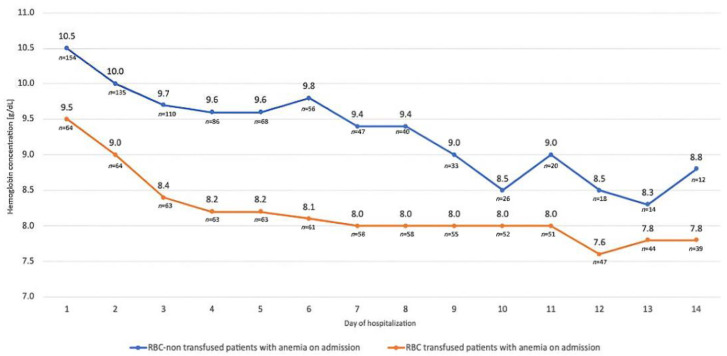
Hemoglobin concentration during first 14 days of hospitalization in patients with anemia on admission with regard to a need for RBC transfusion.

Hemoglobin concentration during the first 14 days of hospitalization in patients with anemia on admission with regard to a need for RBC transfusion is presented in Figure 1.

All daily Hb concentrations in anemic (ICU admission) patients who required RBC transfusion, compared to patients who did not require transfusion, were significantly lower (for all comparisons *p* < 0.05).

**Figure 2:**
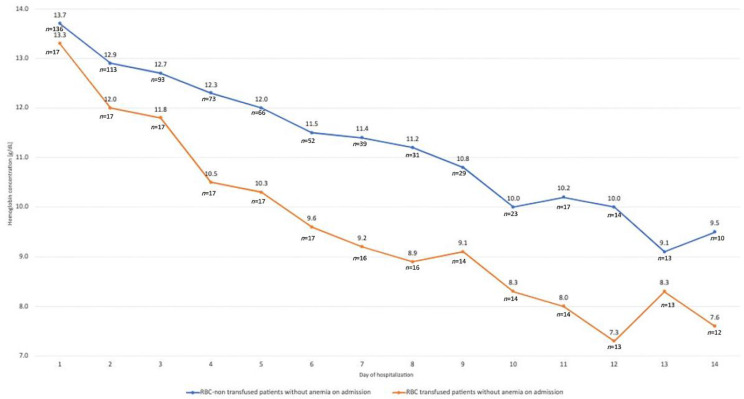
Hemoglobin concentration during first 14 days of hospitalization in patients without anemia on admission with regard to a need for RBC transfusion.

Hemoglobin concentration during first 14 days of hospitalization in patients without anemia on admission with regard to a need for RBC transfusion is presented in Figure 2.

Daily Hb concentrations in non-anemic (ICU admission) patients who required RBC transfusion, compared to patients who did not require transfusion, were significantly lower from day 3 (for all comparisons *p* < 0.05).

Average decrease in Hb during the first week of ICU hospitalization in patients with and without anemia on admission was 1.2 (IQR 0.2–2.3) and 2.8 (IQR 1.1–3.8) g/dL, respectively (*p* < 0.01). Average decrease in Hb during the first week of ICU hospitalization in patients who did not require RBC transfusion was 1.4 (IQR 0.4–2.4) g/dL, and in patients who required RBC transfusion, it was 2.2 (IQR 1.0–3.4) g/dL, leaving patients with Hb at 10.3 (IQR 9.2–12.0) and 8.2 (IQR 7.4–9.1) g/dL, respectively.

Changes in Hb concentration between day 1 and 7 of ICU hospitalization in patients with and without anemia on admission to ICU were not associated with severity of disease (SAPS II, APACHE II, SOFA) (Table 3).

Data on invasive procedures having potential impact on blood loss are presented in Table 4.

Bleeding was more frequent at the insertion site of dialysis catheter (13.3% of patients) and TPE catheter (23.8% of patients) than central venous catheter (7.8% of patients). Bleeding at the insertion site of TPE dialysis catheter occurred in almost one quarter of patients. From all invasive devices inserted in our study population, bleeding at the insertion site of percutaneous tracheostomy was the most common (46.7% of patients with tracheostomy). Extracorporeal circuit clotting occurred in 17.7 and 9.5% of patients undergoing dialysis and TPE, respectively. There were no differences in Hb changes during ICU hospitalization with regard to the use of CRRT. 

Other factors contributing to iatrogenic blood loss in the study population are presented in Table 5.

Median blood loss for repeated laboratory testing in our study population was 13.7 (IQR 9.9–19.3) mL per patient daily. Median laboratory blood losses in septic and non-septic patients per patient daily were 16.2 (IQR 12.7–23.0) and 11.9 (IQR 7.8–16.6) mL (*p <* 0.01), respectively.

Bleeding into tracheobronchial tree and bloody postoperative drainage were the most frequent sources of IBL in the study population.

There were no differences between patients with and without IBL (factors form Table 4 and Table 5, IBL associated with laboratory testing excluded) in the study population with the exception of sepsis/septic shock prevalence (52.6 vs. 47.4%, *p =* 0.01) and ICU-LOS [12.0 (IQR 5.0–22.5) vs. 4.0 (IQR 2.0–8.0), *p <* 0.01)] (Table 6).

## 4. Discussion

In our study, we performed a detailed analysis of factors contributing to iatrogenic blood loss in the critically ill patients in the ICU. From what we know, there have not been many such detailed descriptions in the past. The analysis was performed in order to strengthen the departmental PBM measures. A number of standard PBM measures have already been introduced in the department, such as a reduction in laboratory parameters orders, the use of the smallest available test tubes, and a closed system for blood withdrawal.

Our study population was characterized by a predicted mortality of 12–33%, depending on the classification system used, whereas observed ICU mortality in our cohort was 40.2%. The reason for this discrepancy could be that predicted mortality was assessed on one occasion only—within 24 h of admission to the ICU. The severity of disease since the moment of the ICU admission could have deteriorated, and predicted mortality was not indicative of the real ICU mortality. 

In our study, we retrieved information on acute organ injuries predisposing to bleeding and standard laboratory tests of coagulation. Acute kidney injury was frequent in our patients, although some researchers did not find renal failure to be a risk factor (independent) for major bleeding in critically ill patients [7]; however, in our patients, only minor episodes of bleeding were analyzed, and patients with major bleeding events were excluded. Nevertheless, these minor bleeding events were important to us, as we thought of the study as a tool to improve PBM in our department. Lauzier et al. listed renal replacement therapy to be a time-dependent predictor of major bleeding in critically ill patients receiving heparin thromboprophylaxis (hazard ratio 1.75; IQR 1.20–2.56) [13]. Both these research groups found thrombocytopenia and prolonged aPTT to be independent risk factors for major bleeding [7,13]. In our study population, platelets and aPTT were within normal values. 

In our study, approx. 60% of patients were anemic on admission to ICU. According to published reports, up to 66% of patients are anemic on admission to ICU [1], and almost all become anemic during the next 72 h of ICU hospitalization [2]. The critical impact of the first 72 h of ICU hospitalization on Hb concentration is also present in our study, where we noted the largest decreases in Hb during the first 3 days of hospitalization. In our study, patients who were transfused with RBC were those who had lower Hb at admission and higher daily Hb concentration drops. Restrictive RBC transfusion strategy should be advocated for this group of patients [14].

In our study, minor bleeding in the ICU patients was a frequent occurrence. In patients with percutaneous tracheostomy, almost half of patients bled at the insertion site. Bleeding was also the most common complication of tracheostomy in the current report [15]. Nevertheless, some previous studies reported bleeding complications at 0.6–5% [16]. There is no evidence that bleeding differs with the technique of performing tracheostomy [17]. Due to high prevalence of bleeding from the insertion site of percutaneous tracheostomy, special attention should be paid to prepare the patient for a procedure (coagulation abnormalities, timing of antiplatelet and anticoagulant medications) and meticulous technique. In patients with dialysis catheters, bleeding at the insertion site occurred in approximately 24% (UFH TPE) and 13% (RCA CRRT) of patients. These bleeding episodes may be more frequent. In the study by Arnold et al., 90% of patients experienced bleeding, of which 95% was minor and 5% major [7]. Arnold at al. did not find prophylactic anticoagulation to be an independent risk factor for major bleeding in the critically ill [7]. In our study, therapeutic anticoagulation (UFH) could have impacted on the frequency of bleeding from insertion sites of dialysis catheters inserted to perform TPE. Therapeutic heparin anticoagulation was found to be a significant risk factor for major bleeding in the critically ill [13].

In our study, iatrogenic blood loss for laboratory testing was approx. 13 mL/patient/day. It is slightly higher than in the study by Tosiri at al., who reduced mean drawn blood volume to 9.8 mL/patient/day [18]. Although there was a positive correlation between organ dysfunction and the blood loss at about 41 mL/patient/day in the SOAP study [1], we did not find such correlation. The iatrogenic blood loss in our study was almost one third of that in the study by Vincent et al. 

In our study, bleeding into tracheobronchial tree and postoperative drainage occurred frequently, moderately frequently from gastrointestinal tract and surgical wound, and rarely from genitourinary system and into pleural cavity. Arnold et al. showed that the insertion site of a vascular catheter was the most common location of bleeding (38.3%). Then, bleeding most often came from the endotracheal tube (16.0%), surgical site (14.8%), and skin (12.9%) [7]. The gastrointestinal bleeding in our cohort occurred in 8.6% of patients and was more than two-fold more frequent than in the study by Cook et al. (3.5%); however, Cook et al. analyzed only clinically important gastrointestinal bleeding in mechanically ventilated ICU patients [19].

In our study, we did not find a correlation between a drop in Hb concentration and severity of disease. There were also no differences in Hb drops between groups with regard to the use of CRRT. Moreover, there were no differences in severity of disease between patients with and without IBL. These findings show that Hb drops were not associated with severity of disease.

### Limitations

Although we performed a retrospective study, the collection of bleeding-related data in such a fashion is still possible, as was shown in the prospective study by Arnold et al., where weekend data were collected retrospectively [7]. Our only sources of information were electronic health records, as there was no bleeding reporting tool in use at the time in our ICU. We performed our study in a single ICU in a single institution; therefore, the results obtained may not reflect iatrogenic blood loss in other ICUs. However, by performing this study, we aimed at improving our practice in the context of PBM. Although we did record number of patients in whom bleeding occurred, we did not record the number of bleeding events, in particular patients, its severity, duration, direct consequences, and management. Collection of these detailed bleeding-related data was impossible, as the study was retrospective and covered events that occurred even 2 years before. It was impossible to include information about blood-related skin changes and their size. Moreover, some patients could lose blood during procedures performed outside the ICU (e.g., surgical procedures), which we did not account for. Additionally, we did not investigate the positive or negative impact of medications on Hb concentration [18]. As far as the temporal trend of Hb concentration was concerned, only 73 (19.7%) patients were hospitalized for 14 days, and the rest of the patients were discharged or died earlier. When analyzing the Hb trend, we did not account for fluid balance. Fluid balance may have an impact on Hb concentration, and simple de-resuscitation may increase Hb concentration. 

## 5. Conclusions

Anemia is highly prevalent among medical and surgical patients on admission to ICU and is associated with RBC transfusion. Patients who required RBC transfusion had significantly lower daily Hb concentrations. Severity of disease did not seem to have an impact on Hb concentration. IBL associated with invasive devices and extracorporeal therapies is frequent in ICU patients and may lead to a gradual decrease in Hb concentration. Further studies are required to analyze causes of HAA in the ICU. 

## Figures and Tables

**Table 1:** Study population characteristics.

Characteristic	Value
Sex (female/male) [*n*, %]	168 (45.3)/203 (54.7)
Age (female/male), median, IQR ^1^ [years]	65 (53–72)/65 (55–82)
Sepsis/septic shock [*n*, %]	175 (47.2%)
Patients with acute injuries:	
-acute kidney injury [*n*, %]	153 (41.2)
-acute liver injury [*n*, %]	62 (16.7)
Severity of disease:	
-SAPS II ^2^, median, IQR [points]	44 (31–59)
-APACHE II ^3^, median, IQR [points]	18 (12–24)
-SOFA ^4^, median, IQR [points]	9.0 (5–12)
ICU ^5^ length of stay, median, IQR [days]	6.0 (3.0–14.0)
ICU mortality [*n*, %]	150 (40.2%)

^1^ Interquartile range; ^2^ Simplified Acute Physiology Score II; ^3^ Acute Physiology and Chronic Health Evaluation; ^4^ Sepsis-related Organ Failure Assessment; ^5^ intensive care unit.

**Table 2:** Selected laboratory parameters in patients with and without anemia on admission to ICU.

Parameter	All Patients (Median, IQR ^1^)	Anemia on Admission (*n* = 218) (Median, IQR)	No Anemia on Admission (*n* = 153) (Median, IQR)	*p*
Hb ^2^ [g/dL]	11.6 (9.8–13.3)	10.2 (9.1–11.2)	13.6 (12.7–15.2)	<0.01
Hct ^3^ [%]	34.9 (29.9–40.1)	30.6 (27.8–34.1)	41.0 (37.4–45.0)	<0.01
RBC ^4^ [x 106/µL]	3.9 (3.3–4.4)	3.4 (3.04–3.83)	4.5 (4.2–5.0)	<0.01
MCV ^5^ [fL]	90.2 (86.4–94.6)	89.5 (85.7–94.4)	91.0 (87.6–94.7)	0.20
MCH ^6^ [pg]	30.1 (28.8–31.6)	29.9 (28.4–31.1)	30.5 (29.1–31.8)	0.01
MCHC ^7^ [g/dL]	33.3 (32.1–34.2)	33.1 (31.7–34.0)	33.8 (32.7–34.3)	<0.01
RDW ^8^ [%]	14.5 (13.4–16.1)	15.3 (13.7–17.2)	13.7 (12.8–14.8)	<0.01
RDW-SD ^9^ [fL]	48.4 (43.8–53.0)	49.9 (46.0–55.8)	45.9 (42.2–49.8)	<0.01
Ferritin (*n* = 71) [ng/mL]	773.1 (300.1–1599.5)	795.6 (428.5–1760.9)	646.9 (179.4–1391.0)	0.30
Iron (*n* = 69) [µg/dL]	32.0 (16.7–69.5)	32.0 (14.3–62.9)	36.7 (18.1–102.0)	0.21
Transferrin (*n* = 69) [mg/dL]	136.2 (96.4–182.1)	119.8 (90.8–158.6)	182.8 (151.7–205.9)	<0.01
TS ^10^ (*n* = 69) [%]	16.7 (7.9–33.5)	17.6 (8.1–31.7)	16.6 (7.5–55.7)	0.99
Fibrinogen [mg/dL]	447.0 (300.0–619.7)	481.5 (333.0–650.0)	403.0 (271.3–561.5)	0.01
D-dimers [ng/mL]	4139.5 (1818.0–7473.5)	5107 (2265.5–7617.5)	2430.0 (1327.0–7341.0)	<0.01
Thrombin time [s]	17.7 (15.8–20.8)	17.8 (15.9–20.9)	17.7 (15.7–21.2)	0.90
aPTT [s]	35.1 (29.6–41.4)	36.7 (30.1–43.1)	32.3 (27.8–38.8)	<0.01
Prothrombin time [s]	14.1 (12.7–16.7)	15.0 (13.0–17.8)	13.2 (12.5–15.2)	<0.01
INR	1.2 (1.1–1.5)	1.3 (1.1–1.6)	1.2 (1.1–1.3)	<0.01
Platelets [× 103/µL]	228.0 (165.0–306.2)	212.5 (141.0–318.0)	242.0 (183.8–296.3)	0.03

^1^ Interquartile range; ^2^ hemoglobin; ^3^ hematocrit; ^4^ red blood cell; ^5^ mean cell volume; ^6^ mean cell hemoglobin; ^7^ mean cell hemoglobin concentration; ^8^ red blood cell distribution width; ^9^ red blood cell distribution width-standard deviation; ^10^ transferrin saturation.

**Table 3:** Association between Hb changes during the first 7 days of ICU hospitalization and severity of disease in patients with and without anemia on admission to ICU.

Severity of Disease	Correlation Coefficient	*p*
All patients:		
SAPS II ^1^	−0.10	0.20
APACHE II ^2^	−0.04	0.65
SOFA ^3^	−0.13	0.09
Non-anemic patients:		
SAPS II	−0.11	0.43
APACHE II	−0.07	0.58
SOFA	−0.16	0.23
Anemic patients:		
SAPS II	−0.04	0.66
APACHE II	−0.05	0.58
SOFA	−0.09	0.38

^1^ Simplified Acute Physiology Score II; ^2^ Acute Physiology and Chronic Health Evaluation; ^3^ Sepsis-related Organ Failure Assessment.

**Table 4:** Invasive procedures and iatrogenic blood loss.

Procedure	Value
*Continuous renal replacement therapy*	
-number of patients [*n*, %]	113 (30.5)
-duration, median, IQR [days]	4 (2–8)
-circuit clotting [*n*, %]	20 (17.7)
-bleeding at catheter insertion site [*n*, %]	15 (13.3)
*Therapeutic plasma exchange*	
-number of patients [*n*, %]	21 (5.7)
-circuit clotting [*n*, %]	2 (9.5)
-bleeding at catheter insertion site [*n*, %]	5 (23.8)
*Central venous catheter*	
-number of patients [*n*, %]	257 (69.3)
-bleeding at insertion site [*n*, %]	20 (7.8)
*Tracheostomy*	
-number of patients [*n*]	60 (16.2)
-episodes of bleeding at insertion site [*n*, %]	28 (46.7)
*Percutaneous endoscopic gastrostomy*	
-number of patients [*n*]	42 (11.3)
-bleeding at insertion site [*n*, %]	4 (9.5)

IQR—interquartile range.

**Table 5:** Other factors contributing to iatrogenic blood loss.

Factor	Value
Number of test tubes per patient daily [*n*, IQR ^1^]:	
-arterial blood gas (volume 1.0 mL)	2.0 (1.9−2.4)
-biochemistry (2.5 mL)	1.0 (0.9−1.2)
-complete blood count (2.0 mL)	1.0 (0.7−1.1)
-blood culture (10 mL)	0.6 (0.2−1.0)
-coagulation (2.7 mL)	0.3(0.2−0.7)
Pts ^2^ with bleeding from tracheobronchial tree [*n*, %]	66 (17.8)
Pts ^2^ with bloody postoperative drainage [*n*, %]	65 (17.5)
Pts ^2^ with gastrointestinal bleeding [*n*, %]	32 (8.6)
Pts ^2^ with bleeding from surgical wound [*n*, %]	29 (7.8)
Pts ^2^ with genitourinary bleeding [*n*, %]	20 (5.4)
Pts ^2^ with pleural bleeding [*n*, %]	11 (3.0)

^1^ Interquartile range ^2^ Patients.

**Table 6:** Characteristics of patients with and without iatrogenic blood loss.

Characteristic	Patients with IBL Value	Patients without IBL Value	*p*
Sex (female/male) [*n*, %]	72 (42.6)/97 (57.4)	96 (47.5)/106 (53.5)	0.34
Age (female/male), median, IQR ^1^ [years]	67 (55–71.5)/65 (54.8−73.3)	64.5 (49.5−72)/65 (55.0−71.0)	0.41/0.71
Sepsis/septic shock [*n*, %]	92 (52.6)	83 (47.4)	0.01
Patients with acute injuries:			
-acute kidney injury [*n*, %]	74 (36.6)	128 (63.4)	<0.05
-acute liver injury [*n*, %]	32 (51.6)	30 (48.4)	0.05
Severity of disease:			
-SAPS II ^2^, median, IQR ^1^ [points]	45.0 (32.0−59.0)	43.0 (29.0−58.0)	0.35
-APACHE II ^3^, median, IQR ^1^ [points]	18.0 (13.0−24.3)	18.0 (12.0−23.0)	0.32
-SOFA ^4^, median, IQR ^1^ [points]	9.0 (6.0−12.0)	8.0 (5.0−12.0)	0.11
ICU ^5^ length of stay, median, IQR ^1^ [days]	12.0 (5.0–22.5)	4 (2.0–8.0)	<0.01
ICU mortality [*n*, %]	73 (48.7)	77 (51.3)	0.05
Hb drop 1–7, median, IQR ^1^ [g/dL]	1.8 (0.6–3.1)	1.3 (0.3–2.1)	0.08

^1^ Interquartile range; ^2^ Simplified Acute Physiology Score II; ^3^ Acute Physiology and Chronic Health Evaluation; ^4^ Sepsis-related Organ Failure Assessment; ^5^ intensive care unit.

## Data Availability

The data presented in this study are available on request from the corresponding author.
